# Editorial: Human microbiota: A key player in the etiology and pathophysiology of cardiovascular and metabolic diseases

**DOI:** 10.3389/fcvm.2022.1081722

**Published:** 2022-12-05

**Authors:** Ganesan Velmurugan, Tharmarajan Ramprasath, Gilles Mithieux

**Affiliations:** ^1^Chemomicrobiomics Laboratory, Department of Biochemistry and Microbiology, KMCH Research Foundation, Coimbatore, Tamil Nadu, India; ^2^Center for Molecular and Translational Medicine, Georgia State University, Atlanta, GA, United States; ^3^Centre National de la Recherche Scientifique (CNRS), Paris, France

**Keywords:** microbiota, gut bacteria, metabolic diseases, atherosclerosis, cardiovascular disease, human microbiota

The human microbiota is equivalent to the major organs of the adult human body like the brain and liver, in terms of weight (~1.5 kg) and functionality. The gut, oral cavity, skin, and vagina are major residing homes of the human microbiota. The human microbiota comprises bacteria, viruses, archaea, fungi, and protozoans. Recent studies have revealed the presence of functional microbiota in blood, atherosclerotic plaques, adipose tissues, and other organs. Microbiota and their metabolites play a key role in insulin resistance, atherosclerotic plaque formation, inflammation, oxidative stress, and the metabolism of drugs and chemicals (1). Increasing evidence indicates their key role in the etiology and pathophysiology of different cardiovascular diseases and metabolic diseases. This Research Topic aimed to cover the recent findings on the role of gut microbiota during cardiovascular and metabolic diseases.

The metabolic risk factors, including diabetes, hypertension, and hypercholesterolemia in combination with genetics and lifestyle factors contribute to the etiology of cardiovascular diseases. Guo et al. conduct a systematic review of human observational studies interlinking gut microbiota and hypertension. The database search, as per PRISMA guidelines, resulted in 17 studies with 9,085 participants. The comprehensive analysis of all studies shows gut microbial dysbiosis with decreased diversity and changes in microbial metabolites, specifically short-chain fatty acids, during hypertension.

Yet another major risk factor for cardiovascular diseases is diabetes, which is characterized by an increase in blood glucose. To investigate the role of gut microbiota on the regulation of blood glucose during heart failure, Bao et al. employed a mice heart failure model by performing thoracic aortic constriction followed by an antibiotic cocktail treatment to deplete the microbiota. The microbiota-depleted heart failure mice model showed decreased glucose intolerance and glucagon levels. The changes in gut microbial structure were studied by sequencing and metabolomics. Further, fecal microbiota transplantation experiments reveal the role of microbiota in mediating blood glucose metabolism during heart failure in mice models.

Uric acid is an unexplored marker for heart failure and gut microbiota involves in the metabolism of purines and uric acids (2). In order to validate the potential of serum uric acid as a prognostic marker for heart failure with reduced ejection fraction (HFrEF), Wang et al. investigate the association between hyperuricemia and heart failure parameters in the participants of two large prospective cohort studies. The analysis reveals the association of hyperuricemia with left ventricular ejection fraction recovery and long-term adverse events of heart failure.

Studies have documented the variation in gut microbiota between heart failure patients and healthy controls but fewer studies have attempted to investigate the variation within patients with cardiovascular disease categorized based on any clinical parameters. Heart failure with preserved ejection fraction (HFpEF) is becoming predominant among heart failure conditions. Hence, Huang et al. conduct a preliminary study to understand the variation in gut microbiota during HFpEF. The study includes 30 patients with HFpEF and 30 healthy individuals and gut microbiota profiling. Although no changes in alpha diversity were noticed, a significant variation of microbiota was observed in beta diversity analysis. The authors discuss the probable role of significantly differentially expressed bacteria on host inflammation and metabolic factors.

Atherosclerosis is the thickening of the blood vessel that aids different cardiovascular diseases. Aside from the different molecular pathways proposed for the formation of atherosclerotic plaques, recent research also suggests the role of microbial metabolites in this process (3). The blood acts as an arsenal of numerous microbial metabolites that drives cardiac function and pathology (4). Focusing particularly on plaque stability, Shen et al. review its relationship with gut microbiota. A wide range of data on different microbial metabolites such as short-chain fatty acids, trimethylamine-N-oxide, and their molecular pathway leading to thrombus formation are discussed. In addition, the future therapeutic perspectives based on intervention in microbiota are elaborately presented.

Dietary exposure, environment, and host genetics are the key players in shaping the microbiota of a human (5). The impact of food constituents on gut microbiota is well-established and easily modifiable and thereby provides the scope of dietary-mediated gut microbial restoration as a therapeutic approach. Yang et al. employed the *in vitro* and mice models to investigate the impact of Chinese rice wine metabolites on gut microbiota and subsequent impacts on diabetic cardiomyopathy. The mice treated with rice wine polyphenols and polypeptides led to a proliferation of *Akkermansia* and depletion of *Desulfovibrio* genera. A significant correlation was observed between the cardiac markers and microbiota profile, indicating the microbiota-mediated therapeutic impact.

The gut microbiota interacts with numerous host factors and thereby influences host physiology and pathology. Numerous studies have revealed the role of microRNAs (miRNAs), the small, non-coding RNAs of mammalian origin that negatively regulate gene expression at the post-transcriptional stage. During the past decade, several studies have revealed the role of gut microbiota and miRNAs in different cardiovascular diseases (6). Ionescu et al. provide a comprehensive review of the studies related to microbiota and miRNAs and propose the mechanisms for the synergism between gut microbiota and host miRNAs during cardiovascular diseases.

Recent studies explored the presence of microbiota in traditionally considered sterile tissues such as the liver, pancreas, breast tissue, and bone in both healthy and cancer tissues (7). In continuation of this exploration, Shanmuganathan et al. investigate the variation in the microbiota of human intervertebral disc tissue of healthy donors and patients with cardiovascular diseases undergoing surgery. The study reveals the abundance of proteobacteria (particularly the *Pseudomonas* genus) and firmicutes phyla in the degenerated disks. There is a significant loss in microbial diversity with an abundance of pathogenic bacteria in the degenerated disks.

Overall, this Research Topic provides different dimensions on the role of human microbiota during cardiovascular and other metabolic diseases. The major drawback in microbiota and disease studies is the non-exploration of the chicken and egg hypothesis, i.e., the cause and effect. At this moment, further studies are needed to explore the etiology of changes in the microbiota. We are hopeful that microbiota-based therapeutic approaches discussed here including fecal microbiota transplantation, probiotics, and prebiotic intervention with plant-derived metabolites will evolve into routine clinical practice in the near future.

## Author contributions

All of the contributors contributed to the Editorial process and approved this Editorial article.

## References

[1] Fan Y Pedersen O. Gut microbiota in human metabolic health and disease. Nat Rev Microbiol. (2021) 19:55–71. 10.1038/s41579-020-0433-9 32887946

[2] Wannamethee SG Papacosta O Lennon L Whincup PH. Serum uric acid as a potential marker for heart failure risk in men on antihypertensive treatment: the British Regional Heart Study. Int J Cardiol. (2018) 252:157–92. 10.1016/j.ijcard.2017.11.083 29208425 PMC5766825

[3] Asada Y Yamashita A Sato Y Hatakeyama K. Pathophysiology of atherothrombosis: mechanisms of thrombus formation on disrupted atherosclerotic plaques. Pathol Int. (2020) 70:309–22. 10.1111/pin.12921 32166823 PMC7317428

[4] Velmurugan G Dinakaran V Rajendhran J Swaminathan K. Blood microbiota and circulating microbial metabolites in diabetes and cardiovascular diseases. Tr Endocrinol Metab. (2020) 31:835–47. 10.1016/j.tem.2020.01.013 33086076

[5] Qin Y Havulinna AS Liu Y Jousilahti P Ritchie SC Tokolyi A. Combined effects of host genetics and diet on human gut microbiota and incident disease in a single population cohort. Nat Gen. (2022) 54:134–42. 10.1101/2020.09.12.20193045 35115689 PMC9883041

[6] Li M Chen W Wang Y. The roles of the gut microbiota–miRNA interaction in the host pathophysiology. Mol Med. (2020) 26:101. 10.1186/s10020-020-00234-7 33160314 PMC7648389

[7] Nejman D Livyatan I Fuks G Gavert N Zwang Y Geller LT. The human tumor microbiome is composed of tumor type–specific intracellular bacteria. Science. (2020) 368:6494. 10.1126/science.aay9189 32467386 PMC7757858

